# Design, Dynamics, and Optimization of a 3-DoF Nonlinear Micro-Gyroscope by Considering the Influence of the Coriolis Force

**DOI:** 10.3390/mi13030393

**Published:** 2022-02-28

**Authors:** Sai Wang, Linping Lu, Kunpeng Zhang, Shuying Hao, Qichang Zhang, Jingjing Feng

**Affiliations:** 1 Tianjin Key Laboratory for Advanced Mechatronic System Design and Intelligent Control, School of Mechanical Engineering, Tianjin University of Technology, Tianjin 300384, China; ws1546641097@163.com (S.W.); jjfeng@tju.edu.cn (J.F.); 2 National Demonstration Center for Experimental Mechanical and Electrical Engineering Education, Tianjin University of Technology, Tianjin 300384, China; 3 Tianjin Key Laboratory of Nonlinear Dynamics and Control, School of Mechanical Engineering, Tianjin University, Tianjin 300072, China; qzhang@tju.edu.cn

**Keywords:** complete 2-DOF sense mode, nonlinear micro-gyroscope, bandwidth, gain, dynamical characteristics

## Abstract

In this paper, we use the nonlinear hardening stiffness of drive mode deal with the contradiction between gain and bandwidth of the linear micro-gyroscope, to improve the bandwidth and gain in sense direction. Firstly, in order to adjust the distance between two resonant peaks, we changed an incomplete two-degree-of-freedom(2-DoF) sense mode system of the micro-gyroscope into a complete 2-DoF system. Afterward, according to the given nonlinear coefficient of stiffness of drive mode, the structure size of driving micro-beams was designed to obtain a nonlinear micro-gyroscope with controllable stiffness. Finally, we investigated the effects of peaks spacing, damping, and driving nonlinearity on gain and bandwidth, and the nonlinear micro-gyroscope was optimized by orthogonal experiment method and response surface method. The results reveal that the peaks spacing has a great influence on the gain and bandwidth of both linear and nonlinear micro-gyroscopes. The larger the peaks spacing, the lower the gain, but higher gain can be achieved when the resonant frequency of the drive mode is close to the lower-order resonant frequency of the sense mode. Driving nonlinearity leads to the response peak of the Coriolis force to have a hardening characteristic, thus forming a wide platform in the sense direction. Hardening of the response peak of the Coriolis force allows the micro-gyroscope to obtain a higher gain while the bandwidth of the sense mode is also greatly improved. In addition, parameter optimization can make the gain and bandwidth of the micro-gyroscope optimal. When the peaks spacing is small and the nonlinear stiffness coefficient is about 10^12.2^, under the premise that the gain is basically constant, the bandwidth of the sense mode increases about 1.76 times compared with the linear gyroscope. Damping can suppress the influence of nonlinearity in a micro-gyroscope system. Within a certain range, the frequency response of the nonlinear micro-gyroscope tends to be a linear system with the increase in damping, resulting in narrower bandwidth and lower gain.

## 1. Introduction

The micro-gyroscope is the key component of inertial measurement unit and inertial navigation system. It is widely used to measure the angular velocity of rotating carriers. Micro-gyroscopes could be applied to many fields, such as robots, airplanes, and missiles in military fields, as well as vehicle navigation, mobile applications, and mobile phone positioning in civilian fields [1].

Gain and bandwidth are the main performance metrics of the micro-gyroscope. Based on the principle of the Coriolis effect, micro-gyroscopes are usually divided into two categories including resonant and non-resonant gyroscopes. In resonant micro-gyroscopes, the device operates at resonance and the resonant frequencies of drive and sense mode are generally matched, which leads to high mechanical gain. However, the bandwidth of the resonant micro-gyroscopes is extremely narrow. Xu et al. [2] studied the characteristics of resonant micro-gyroscopes with different frequency mismatches. Their results showed that the frequency mismatch could lead to a significant decrease in the gain of the sense mode and the frequencies can be matched by adjusting the DC voltage of the sense mode to control the resonant frequency. Generally speaking, the resonant frequencies of the drive and sense mode are equal in the resonant gyroscopes, while the bandwidth is only about 2 Hz.

Non-resonant gyroscopes, i.e., multi-degree-of-freedom (multi-DoF) systems, tackle this problem of narrow bandwidth of resonant gyroscopes at the expense of the gain. In the research of non-resonant gyroscopes, researchers often ignore the influence of the Coriolis force on the frequency response of the sense mode for convenience. Bukhari et al. [3] investigated a multi-DoF gyroscope with an incomplete 2-DoF sense mode system. On the one hand, the results revealed the advantages of large resonant peaks spacing and wide bandwidth. On the other hand, it also revealed the shortcomings of the incomplete 2-DoF system, i.e., the gain is low and the peaks spacing cannot be adjusted. In order to realize the arbitrary adjustment of the resonant peaks spacing, Esmaeili et al. [4] explored a single-DoF drive mode and two-DoF sense mode (SD-TD) gyroscope. They changed the incomplete 2-DoF system into a complete 2-DoF system and arranged two operational regions of the micro-gyroscope, i.e., wide-bandwidth low-gain region and high-gain narrow-bandwidth region. It is well known that the frequency of the Coriolis force of the SD-TD gyroscope is equal to the resonant frequency of the drive mode. There are not only two peaks corresponding to the resonant frequency of the sense mode on the frequency response, but also a response peak corresponding to the frequency of the Coriolis force. This response peak of the Coriolis force directly affects the bandwidth and gain in sense direction. Therefore, ignoring the influence of the Coriolis force cannot correctly reflect the true performance of the micro-gyroscope. Verma et al. [5] investigated a SD-TD gyroscope by considering the effect of the Coriolis force, and the frequency response of the sense mode was divided into two bandwidths. Hao et al. [6] decoupled the SD-TD to reduce the same frequency coupling of the drive and sense modes and discussed the influence of nonlinear hardening stiffness of the drive mode on the frequency response of the sense mode. The results showed that the peak induced by the Coriolis force tended to harden when the Coriolis force was considered. In this case, the nonlinearity affected the choice of bandwidth. Therefore, considering the influence of Coriolis force is an important aspect in the analysis of a micro-gyroscope. 

Recently, it has been indicated that there are many nonlinear factors in the micro-electromechanical system (MEMS), such as stiffness nonlinearity, material nonlinearity, crack nonlinearity, and electrostatic forces nonlinearity. The existence of these nonlinear factors may bring some negative effects. Larkin et al. [7] considered the nonlinearity caused by cracked beams. Severe single or multiple cracks significantly reduced the gain of the micro-gyroscope, resulting in the performance degradation of the MEMS sensors. Yoon et al. [8] investigated a ring resonator micro-gyroscope by considering the damping and electrostatic force nonlinearity. Their results showed that the nonlinearity generates high-order coupling terms and cannot be neglected. High frequency vibration may excite the flexural modes, leading to undesired responses that cannot be distinguished from the desired responses excited by the operation of the ring gyroscope.

It is worth noting that utilizing nonlinear characteristics to improve the performance of micro-machines has attracted a lot of attention. The unique softening and hardening of nonlinear dynamic systems could be expected to overcome the disadvantages of linear systems. Ghasemi et al. [9] used the L-shaped beam with nonlinear softening stiffness to increase the bandwidth and gain of the energy harvester. The acquisition effect of the energy harvester by considering both the electrostatic force nonlinearity and the stiffness softening nonlinearity has also been discussed. Adhikari et al. [10] designed a nonlinear mass sensor by combining a cantilever beam and a nonlinear cosine beam. Utilizing the characteristic of nonlinear amplitude jump, the detected mass was determined by the frequency difference before and after the measurement, which greatly improved the detection gain. Hao et al. [11] analyzed a multi-DoF gyroscope with both electrostatic nonlinearity and stiffness hardening nonlinearity. Hardening characteristics of the micro-beams were used to balance the softening characteristics caused by electrostatic force. Finally, the frequency response presented an excellent linear state. Radgolchin et al. [12] considered the influence of nonlocal and gradient strains to study the nonlinear vibration characteristics of a beam gyroscope. Their results showed that the pull-in instability was delayed, and the stable operating ranger would be broader by applying the electrostatic loads. Using nonlinear dynamics to improve the performance of energy harvester and MEMS sensors has become a research hotspot in recent years. It also provides a possible way to overcome the inherent shortcomings of linear micro-gyroscopes with restricting gain and bandwidths. 

For the time being, there are few studies on utilizing nonlinearity to improve the dynamic performance of micro-gyroscopes. In this paper, based on the reference [6], we improved an incomplete 2-DoF sense system of a micro-gyroscope to a complete 2-DoF system for adjusting the distance between two resonant peaks of the sense mode. We discussed the influence of peaks spacing and damping on the bandwidth of the sense mode. Gain and bandwidth of every peaks spacing were optimized by response surface method. According to the given nonlinear stiffness coefficient, the structure size of the driving micro-beam was designed, and a nonlinear micro-gyroscope with controllable stiffness was obtained. The effect of nonlinear stiffness on gain and bandwidth are also investigated.

## 2. Parameter Model of the Micro-Gyroscope and Its Mathematical Description

**Figure 1:**
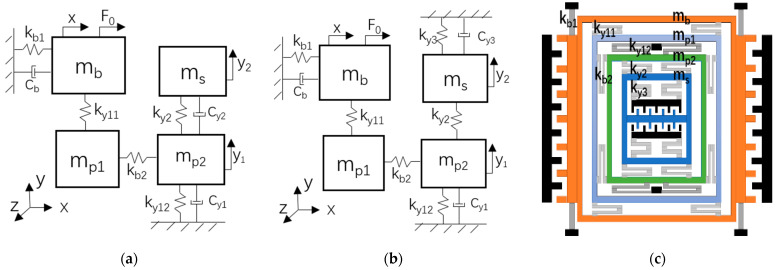
Fully decoupled 3-DoF micro-gyroscope: (**a**) Incomplete 2-DoF sense mode system; (**b**) Complete 2-DoF sense mode system; (**c**) Physical schematic diagram of the micro-gyroscope.

Figure 1a is the lumped parameter model of a decoupled 3-DoF micro-gyroscope, where the *x*-axis represents the drive direction. When a harmonic excitation $F_{0}$ acts on the mass $m_{b}$, the masses $m_{p1}$ and $m_{b}$ vibrate along the *x*-axis together and form a single-DoF system. In the sense direction of the *y*-axis, the mass $m_{p1}$ and $m_{p2}$ constitute the first DoF, which is defined as sense-I; the mass $m_{s}$ is the second DoF, which is defined as sense-II. By these means, we construct a 3-DoF micro-gyroscope with a drive mode and two sense modes (sense-I and sense-II). If there is an angular velocity input on the *z*-axis, the masses of sense-I and sense-II vibrate along the *y*-axis because of the Coriolis force. In this process, the mass $m_{p1}$ transfers vibration energy of the drive mode to the sense mode, so the drive and sense masses only vibrate along their respective directions under the constraint of the spring. Then, the decoupling design of the micro-gyroscope is realized. 

Figure 1a describes the dynamical model of the micro-gyroscope with an incomplete 2-DOF sense mode system. An incomplete 2-DOF system means that it is based on the principle of dynamic vibration absorber [3]. In order to realize the arbitrary adjustment of the resonant peaks spacing, a complete 2-DOF sense mode system is constructed by adding a spring $k_{y3}$ as shown in Figure 1b, where, $k_{y11}$, $k_{y12}$, $k_{y2}$, and $k_{y3}$ represent the equivalent spring stiffness of the micro-beams of the sense mode; $c_{y1}$ and $c_{y3}$ represent the damping coefficients of the sense mode. The physical schematic diagram of the micro-gyroscope with a complete 2-DOF sense mode system is described as shown in Figure 1c.

Dynamical equations of the fully decoupled 3-DOF gyroscope are deduced as follows:

(1) $$m_{x}\ddot{x}+c_{b}\dot{x}+k_{b}x=F_{0}\sin\left(w_{0}t\right)$$

(2) $$\begin{array}{l} m_{y1}{\ddot{y}}_{1}+c_{y1}{\dot{y}}_{1}+\left(k_{y1}+k_{y2}\right)y_{1}-k_{y2}y_{2}=-2m_{y1}\Omega_{z}\dot{x}\\ m_{y2}{\ddot{y}}_{2}+c_{y3}{\dot{y}}_{2}+\left(k_{y2}+k_{y3}\right)y_{2}-k_{y2}y_{1}=-2m_{y2}\Omega_{z}\dot{x} \end{array}$$

where $m_{x}$, $c_{b}$, and $k_{b}$ are the mass, damping, and spring coefficients in the drive direction, respectively. $F_{0}$ is the amplitude of the exciting force. $\omega_{0}$ is the frequency of the exciting force and $m_{x}=m_{b}+m_{p1}$, $m_{y1}=m_{p1}+m_{p2}$, $m_{y2}=m_{s}$, $k_{b}=k_{b1}+k_{b2}$, $k_{y1}=k_{y11}+k_{y12}$. Let $x=A_{x}\sin(\omega_{0}t-\varphi )$ express the steady-state solution of Equation (1), and we obtain:

(3) $$A_{x}=\frac{F_{0}}{\left(k_{b}\sqrt{\left({\left(1-\frac{\omega_{0}{}^{2}}{\omega_{x}{}^{2}}\right)}^{2}+4\xi_{x}{}^{2}\frac{\omega_{0}{}^{2}}{\omega_{x}{}^{2}}\right)}\right)}$$

(4) $$\varphi =\arctan\left(\frac{2\xi_{x}\omega_{0}}{\omega_{x}\left(1-\frac{\omega_{0}{}^{2}}{\omega_{x}{}^{2}}\right)}\right),\omega_{x}=\sqrt{\frac{k_{b}}{m_{x}}},\xi_{x}=\frac{c_{b}}{2m_{x}\omega_{x}}$$

where $A_{x}$, φ, $\omega_{x}$, and $\xi_{x}$ are the amplitude, phase, resonant frequency, and damping ratios of the drive mode, respectively.

## 3. Linear Design and Analysis

### 3.1. Design of the Complete 2-DoF Sense Mode System

Since the complete 2-DOF sense mode system is decoupled from the drive mode, we can design it independently. Structure frequencies of the sense mode are set as:

(5) $$\omega_{a}{}^{2}=\frac{k_{y1}+k_{y2}}{m_{y1}},\omega_{b}{}^{2}=\frac{k_{y2}+k_{y3}}{m_{y2}},\omega_{c}{}^{2}=\frac{k_{y2}}{\sqrt{m_{y1}m_{y2}}}$$

In Equation (5), the structure frequencies $\omega_{a}$ and $\omega_{b}$ can be designed independently of the coupling frequency $\omega_{c}$. By these means, it can be resolved that the sense mode peaks spacing cannot be adjusted arbitrarily [3]. Substituting Equation (5) into the eigenvalue equation $\left|\left(\left[K\right]-p^{2}\left[M\right]\right)\right|=0$ of Equation (2), the resonant frequencies of the sense mode can be obtained as:

(6) $$p_{1,2}^{2}=\frac{1}{2}\left(\omega_{a}^{2}+\omega_{b}^{2}\mp \sqrt{{\left(\omega_{a}^{2}-\omega_{b}^{2}\right)}^{2}+{\left(2\omega_{c}^{2}\right)}^{2}}\right)$$

where, $\left[M\right]=\left[\begin{array}{cc} m_{y1} & 0\\ 0 & m_{y2} \end{array}\right]$ and $\left[K\right]=\left[\begin{array}{cc} k_{y1}+k_{y2} & -k_{y2}\\ -k_{y2} & k_{y2}+k_{y3} \end{array}\right]$.

The sense mode of the micro-gyroscope includes sense-I and sense-II. Assuming that the frequency $\omega_{r}$ is located in the middle of the two resonant frequencies of the sense mode, the resonant frequencies of the sense mode are written as $p_{1,2}=\omega_{r}\mp \Delta /2$ and $\Delta =p_{2}-p_{1}$, where Δ is the peaks spacing. Substituting them into Equation (6) and solving the structural frequencies is as follows:

(7) $$\omega_{a,b}{}^{2}=\frac{1}{4}\left(\Delta^{2}+4\omega_{r}^{2}\pm 4\sqrt{-\omega_{c}^{4}+\Delta^{2}\omega_{r}^{2}}\right)$$

Then, $\Delta^{2}\omega_{r}^{2}-\omega_{c}^{4}\geq 0$ is the constraint condition for Equation (7). Introduce the proportional parameter ϵ and $\omega_{c}^{2}=\epsilon \Delta \omega_{r}(0<\epsilon <1)$. We simplify Equation (7) to obtain:

(8) $$\omega_{a,b}{}^{2}=\omega_{r}^{2}+{\left(\frac{\Delta }{2}\right)}^{2}\pm \omega_{r}\Delta \sqrt{1-\epsilon^{2}}$$

Introducing the mass ratio $\mu^{2}=m_{y2}/m_{y1}$, and substituting it and Equation (5) into Equation (8), the stiffness of the sensing micro-beam is given by:

(9) $$\begin{array}{l} k_{y1}=m_{y1}\omega_{a}^{2}-k_{y2},\\ k_{y2}=\epsilon \Delta \mu m_{y1}\omega_{r},\\ k_{y3}=\mu^{2}m_{y1}\omega_{b}^{2}-k_{y2} \end{array}$$

According to the known parameters $m_{y1}$, $m_{y2}$, $\omega_{r}$, and Δ, we can calculate the stiffness coefficient of the sensing micro-beam. Because the choice of value ϵ will affect the gain of the sense mode, explore the value of ϵ that maximizes the gain. Applying the transfer function method to solve the frequency response of sense-II in Equation (2) [13], the result has the form:

(10) $$\frac{B_{2}}{\Omega_{z}}=\left|-\frac{2A_{x}m_{y2}\omega_{0}\left(s\mu c_{y1}+m_{y1}\left(s^{2}\mu +\mu \omega_{a}^{2}+\omega_{c}^{2}\right)\right)}{\nabla (\omega_{0})}\right|$$

where *B*_2_ is the output amplitude of sense-II. In order to analyse the rule of the frequency response of the sense-II with respect to the parameter ϵ, by substituting the Coriolis acceleration $a_{c}=2\Omega_{z}\omega_{0}A_{x}$ into Equation (10), the result will be:

(11) $$\frac{B_{2}}{a_{c}}=\left|-\frac{m_{y2}\left(s\mu c_{y1}+m_{y1}\left(s^{2}\mu +\mu \omega_{a}^{2}+\omega_{c}^{2}\right)\right)}{\nabla (\omega_{0})}\right|$$

where $\nabla (\omega_{0})=sc_{y1}\left(sc_{y3}+m_{y2}\left(s^{2}+\omega_{b}^{2}\right)\right)+m_{y1}\left(sc_{y3}\left(s^{2}+\omega_{a}^{2}\right)+m_{y2}\left(s^{4}+s^{2}\omega_{b}^{2}+\omega_{a}^{2}\left(s^{2}+\omega_{b}^{2}\right)-\omega_{c}^{4}\right)\right)$. Let $c_{y1}=c_{y2}=c_{y3}=0$. Substituting $s=i\omega_{r}$ (i is the imaginary unit) and Equation (8) into Equation (11) to obtain the gain *G*_2_ of sense mode is as follows:

(12) $$G_{2}=-\frac{4\left(\mu \Delta +4\left(\epsilon +\sqrt{1-\epsilon^{2}}\mu \right)\omega_{r}\right)}{\mu \Delta \left(\Delta^{2}-16\omega_{r}^{2}\right)}$$

**Figure 2:**
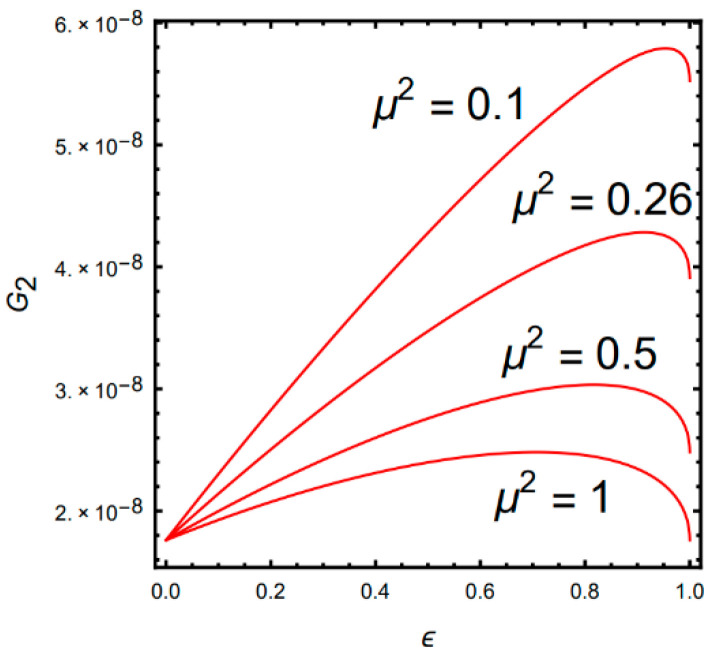
Relationship of gain *G*_2_ and parameter ϵ.

Choosing $f_{r}=5.4\mathrm{kHz}$ and Δ=280Hz, according to Equation (12), the relationship between gain $G_{2}$ and parameter ϵ is shown in Figure 2.

Figure 2 shows that the smaller the mass ratio, the higher the gain. The parameter ϵ has an extreme point which corresponds to the highest gain. The extreme point can be obtained by differentiating Equation (12). In order to ensure the highest gain of the sense mode obtained by Equation (10), the mass ratio $\mu^{2}$ should be selected as small as possible and satisfied as $\epsilon =1/\sqrt{1+\mu^{2}}$ from the differential of Equation (12).

### 3.2. Estimation of Damping

In the micro-gyroscope’s structure, the dominant energy dissipation is slide and squeeze film damping [3]. The damping $c_{b}$ is approximately the combination of the slide film damping between the mass $m_{b}+m_{p1}$ and substrate, together with the slide film damping between the air-gap capacitor fingers. The damping $c_{y1}$ is approximately the sliding film damping between the mass $m_{p1}+m_{p2}$ and substrate. The damping $c_{y3}$ is approximately the combination of the slide film damping between the mass $m_{s}$ and substrate, together with the slide film damping between the air-gap capacitor fingers. 

The calculation formulas of corresponding damping will be [14]:

(13) $$\begin{array}{l} c_{b}={µ}_{e}\frac{A_{b}+A_{p1}}{z_{0}}+{µ}_{e}\frac{2N_{comb}l_{comb}t}{y_{comb}},\\ c_{y1}={µ}_{e}\frac{A_{p1}+A_{p2}}{z_{0}},\\ c_{y3}={µ}_{e}\frac{A_{s}}{z_{0}}+{µ}_{e}\frac{2N_{cap}l_{cap}t}{y_{cap}} \end{array}$$

where the effective viscosity is ${µ}_{e}={µ}_{p}P$, the air viscosity constant is ${µ}_{p}=2.78\times 10^{-6}{\;\mathrm{kg}/m}^{2}.s.\mathrm{Pa}$, and the packaging pressure below standard atmospheric pressure is $P=1.01\times 10^{5}\;\mathrm{Pa}$. $z_{0}$ is the distance between the micro-gyroscope and substrate; $N_{comb}$ and $N_{cap}$ are the number of comb fingers of the drive and sense mode; $l_{comb}$ and $l_{cap}$ are the overlapping length of the comb fingers; and $y_{comb}$ and $y_{cap}$ are the gap between the comb fingers. According to Equation (13), we can calculate the damping coefficient of the micro-gyroscope.

### 3.3. Linear Analysis

In Reference [15], the authors investigated a linear modal analysis on the micro-gyroscope with a complete 2-DoF sense mode, and the theoretical calculation and modal analysis results corresponded well. Schofield et al. carried out a theoretical analysis of this micro-gyroscope and used experiments to verify the theory in [16]. This paper cites the results of the reference [15] and focuses on the bandwidth and gain of the nonlinear micro-gyroscope.

**Figure 3:**
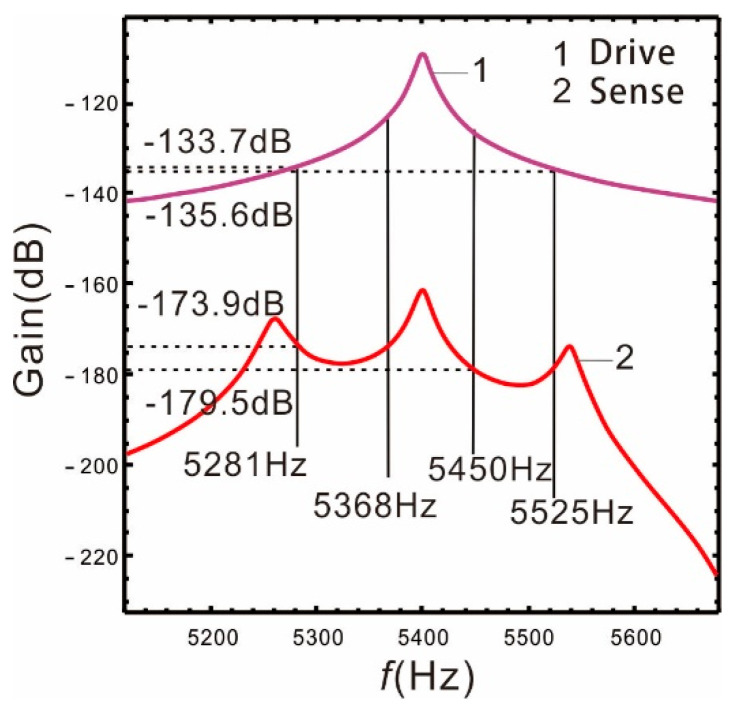
Frequency response of the drive mode and sense mode.

Usually, the definition of bandwidth of the micro-gyroscope is: $S\left(\omega \right)-S\left(\omega_{0}\right)=3$, and $S\left(\omega \right)=20Log\left(B_{2}/\Omega_{z}\right)$. The main parameters are shown in Table 1, and the other parameters are selected as follows: $\omega_{x}=\omega_{r}=5400\times 2\pi \;\mathrm{rad}/s$, P=10 Pa, and Δ=280 Hz. Substituting $s=i\omega_{0}$ into Equations (3) and (10), Figure 3 shows the frequency responses of the drive and sense mode.

In Figure 3, the peaks on both sides of the frequency response of the sense mode correspond to the resonant frequency. The middle peak is produced by the Coriolis force [5] (abbreviated as Coriolis peak), and its occurrence position corresponds to the resonant frequency of the drive mode.

When the Coriolis peak is located in the middle of the two resonant peaks of the sense mode, it has the same influence on the two sense peaks, which is not conducive to broaden the bandwidth of the nonlinear micro-gyroscope. In order to make the resonant frequency of the drive mode close to the low-order resonant frequency of the sense mode, we select the frequencies $\omega_{r}=5330\times 2\pi \;\mathrm{rad}/s\;$ and $\omega_{r}=5400\times 2\pi \;\mathrm{rad}/s$ The peaks spacingis respectively selected as 560 Hz, 280 Hz, and 230 Hz. The relationship of the gain and resonant frequency of the drive and sense mode are shown as follows.

**Figure 4:**
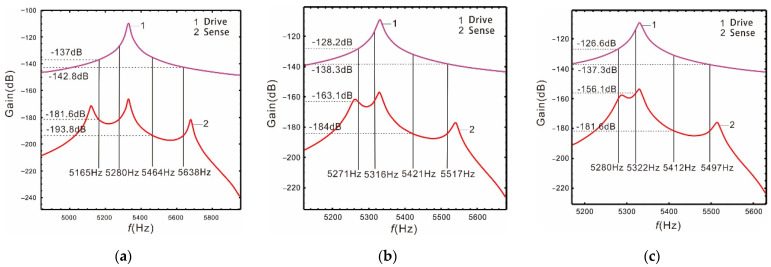
Linear case: the resonant frequency of the drive mode close to the low-order resonant frequency of the sense mode: (**a**) Δ=560Hz; (**b**) Δ=280Hz; (**c**) Δ=230Hz.

As shown in Figure 4, the smaller the distance between the two sense mode resonant peaks, the higher the gain. If the resonant frequency of the drive mode is close to the low-order resonant frequency of the sense mode, the gain near the low-order resonant frequency is higher, but the bandwidth is narrower. Meanwhile, the gain of the drive mode corresponding to its 3dB bandwidth is also higher. It also conforms to the contradiction between bandwidth and gain. Based on the above analysis, we propose to deal with the problem of opposing bandwidth and gain by using the nonlinearity of the straight beam.

## 4. Nonlinear Design and Analysis

### 4.1. Design of Nonlinear Micro-Beam

The force-displacement relationship of a resonator driven by the straight beam is described as [17]:

(14) $$F(x)=\frac{12EI}{L_{1}{}^{3}}x+\frac{18EA}{25L_{1}{}^{3}}x^{3}=K_{Linear}x+K_{Nonlinear}x^{3}$$

where the Young’s modulus is $E=1.69\times 10^{11}$, the section moment of inertia is $I=tb^{3}/12$, the section area is A=tb, *t* is the thickness of the beam, and b is the width of the beam. Since the micro-gyroscope is decoupled, there are eight beams in the driving direction as shown in Figure 1c. $k_{b1}$ is the equivalent stiffness of the four straight beams for the drive mode. $k_{b2}$ is the equivalent stiffness of the four U-shaped beams for decoupling between the drive and sense mode. The total force $F_{0}$ is the sum of the equivalent stiffness of the eight beams and has the following form [18]:

(15) $$F_{0}=\sum_{i=1}^{4}F_{i}=\sum_{i=1}^{4}(K_{Linear}x+K_{Nonlinear}x^{3})=k_{b2}x+k_{b1}x+k_{d}x^{3}$$

where $k_{b1}$ and $k_{d}$ are the linear and nonlinear equivalent stiffness coefficients of the straight beam. According to Equation (14), we have:

(16) $$\frac{K_{Nonlinear}}{K_{Linear}}=\frac{k_{d}}{k_{b1}}=\frac{18}{25b^{2}}$$

The minimum width b of the beam in the micro-gyroscope is 8 µm. If the width of the beam is selected as the minimum value, the maximum of nonlinear coefficient $k_{d}$ is about $10^{12.547}$ in Equation (16). The nonlinear characteristics begin to appear when $k_{d}\geq 10^{11}$ [6]. Therefore, the range of the nonlinear coefficient is $10^{11}\leq k_{d}\leq 10^{12.547}$. The parameters of the straight beam are $k_{b1}=297.54Ν/m$, $b=11.6\times 10^{-6}m$, and the length of the beam is $L=658.6\times 10^{-6}m$. The calculated nonlinear coefficient $k_{d}=10^{12.2}$ is within the required interval.

### 4.2. Approximate Analytical Solution of the Nonlinear Micro-Gyroscope

According to Equation (1), we rewrite the dynamical equation of the drive mode as:

(17) $$\ddot{x}+C_{B}\dot{x}+\omega_{x}^{2}x+K_{D}x^{3}=F\sin\left(w_{0}t\right)$$

where $\omega_{x}^{2}=k_{b}/m_{x}$, $C_{B}=c_{b}/m_{x}=2\xi_{x}\omega^{2}$, $\xi_{x}=c_{b}/2m_{x}\omega_{x}$, $K_{D}=k_{d}/m_{x}$, and $F=F_{0}/m_{x}$. Equation (17) is the forced vibration of a single-DoF damped Duffing system with harmonic excitation. Introducing detuning parameters σ, the approximate periodic response of the primary resonance is analyzed by using the multiple time scales method [19]. The following expression is given:

(18) $$\omega_{0}=\omega_{x}+\varepsilon \sigma ,C_{B}=\varepsilon c_{B},K_{D}=\varepsilon k_{D},F=\varepsilon f$$

(19) $$\begin{array}{l} x(t,\varepsilon )=x_{0}(T_{0},T_{1})+\varepsilon x_{1}(T_{0},T_{1})+O(\varepsilon^{2}),\\ T_{n}=\varepsilon^{n}t;n=0,1,2\ldots \end{array}$$

where ε is a small parameter. Substituting Equations (18) and (19) into (17), Equation (17) can be deduced as:

(20) $$\begin{array}{l} \left(D_{0}^{2}+2\varepsilon D_{0}D_{1}+\varepsilon^{2}D_{1}^{2}\right)\left(x_{0}+\varepsilon \;x_{1}\right)+\omega_{x}^{2}\left(x_{0}+\varepsilon \;x_{1}\right)=\\ -\varepsilon c_{B}\left(D_{0}+\varepsilon D_{1}\right)\left(x_{0}+\varepsilon \;x_{1}\right)-\varepsilon k_{D}{\left(x_{0}+\varepsilon \;x_{1}\right)}^{3}+\varepsilon f\sin(\omega_{0}t) \end{array}$$

where *D_n_* (*n* = 0,1) is the partial differential operator about *T_n_*. Comparing the same order coefficients of ε, the following partial differential equations are given:

(21) $$\varepsilon^{0}:D_{0}^{2}x_{0}+\omega_{x}^{2}x_{0}=0$$

(22) $$\varepsilon^{1}:D_{0}^{2}x_{1}+\omega_{x}^{2}x_{1}=-2D_{0}D_{1}x_{0}-c_{B}D_{0}x_{0}-k_{D}x_{0}^{3}+f\sin(\omega_{0}t)$$

The general solution form of Equation (21) is expressed as $x_{0}=E(T_{1})\exp[i\omega_{x}T_{0}]+cc$, where “*cc*” represents the conjugate of the previous items. Substitute it into Equation (22):

(23) $$\begin{array}{c} D_{0}^{2}x_{1}+\omega_{x}^{2}x_{1}=-[2D_{1}i\omega_{x}E+c_{B}i\omega_{x}E+k_{D}3E^{2}\bar{E}+\frac{if}{2}\exp(i\sigma T_{1})]\exp(i\omega_{x}T_{0})\\ -k_{D}E^{3}\exp(3i\omega_{x}T_{0})+cc \end{array}$$

The average equation of Equation (23) can be deduced by the solvability condition that does not produce a secular term:

(24) $$2D_{1}i\omega_{x}E+c_{B}i\omega_{x}E+k_{D}3E^{2}\bar{E}+\frac{if}{2}\exp(i\sigma T_{1})=0$$

Set $E(T_{1})=1/2A_{x}(T_{1})\exp[i\beta (T_{1})]$ and substitute it into Equation (24):

(25) $$\frac{dA_{x}}{dT_{1}}+A_{x}i\sigma -A_{x}i\frac{d\varphi }{dT_{1}}+\frac{c_{B}A_{x}}{2}-\frac{3ik_{D}A_{x}^{3}}{8\omega_{x}}+\frac{f}{2\omega_{x}}\exp(i\varphi )=0$$

Let $\varphi =\sigma T_{1}-\beta$, $\beta^{\prime }=\sigma -\varphi^{\prime }$, substitute them into Equation (25), and separate the real and imaginary parts:

(26) $$\begin{array}{l} \frac{dA_{x}}{dT_{1}}=-\frac{c_{B}A_{x}}{2}-\frac{f}{2\omega_{x}}\cos(\varphi )\\ A_{x}\frac{d\varphi }{dT_{1}}=A_{x}\sigma -\frac{3k_{D}A_{x}^{3}}{8\omega_{x}}+\frac{f}{2\omega_{x}}\sin(\varphi ) \end{array}$$

Let the right side of Equation (26) equal to zero and eliminate the trigonometric function to get the nonlinear frequency response equation of the drive mode:

(27) $$16{\left(c_{B}A_{x}\omega_{x}\right)}^{2}+{\left(3k_{D}A_{x}^{3}-8\omega_{x}A_{x}\sigma \right)}^{2}=16f^{2}$$

The frequency response is calculated by Equations (1), (2), and (27). The approximate solution is also verified by using Runge—Kutta methods as follows.

**Figure 5:**
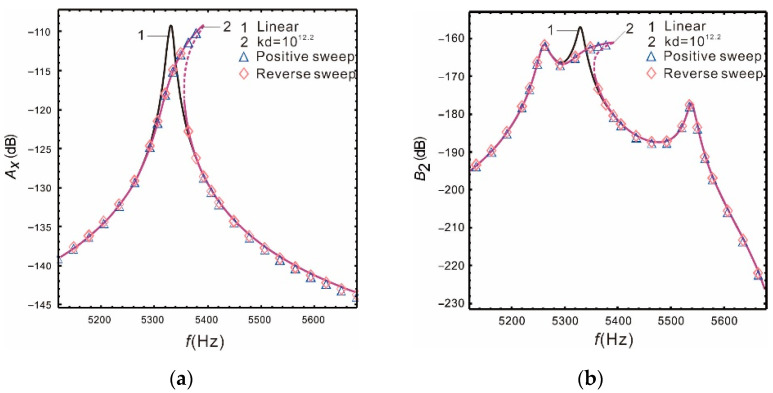
Comparison of Runge—Kutta methods and multiple time scales method: (**a**) drive mode; (**b**) sense mode.

Figure 5 is a comparison of the nonlinear, analytical, numerical, and linear solutions when peaks spacing is  Δ=280 Hz. It is demonstrated that nonlinear hardening occurs in the drive mode and the resonant frequency increases slightly. However, the two resonant peaks of the sense mode are not affected by nonlinearity; the Coriolis peak also has nonlinear hardening, and the peak becomes flatter. With the input of the external angular velocity, both the drive mode response and the Coriolis peak will produce hardening characteristics, which can effectively increase the bandwidth of the micro-gyroscope. Furthermore, it is verified that the nonlinear micro-gyroscope can deal with the conflict between the bandwidth and gain of the linear micro-gyroscope. The above results also show good correspondence between the analytical and the numerical solution.

### 4.3. Nonlinear Analysis

#### 4.3.1. Bandwidth and Gain of Nonlinear Micro-Gyroscopes

**Figure 6:**
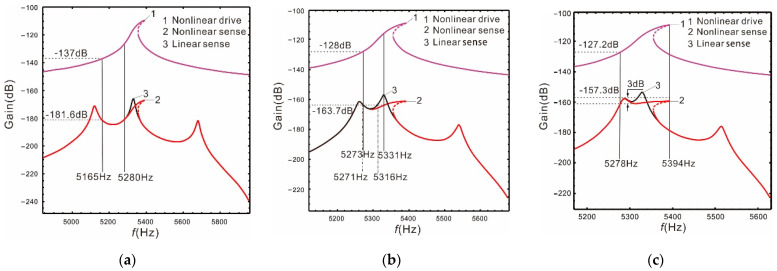
Nonlinear case: the resonant frequency of the drive mode close to the low-order resonant frequency of the sense mode: (**a**)  Δ=560 Hz; (**b**)  Δ=280 Hz; (**c**)  Δ=230 Hz.

It is shown in Figure 6 that the nonlinear (or linear) bandwidth is the distance between the vertical solid (or dotted) lines. If the peaks spacing is as wide as  Δ=560 Hz, the nonlinearity of the Coriolis peak has almost no effect on the bandwidth. If the peaks spacing is  Δ=280 Hz, the nonlinear bandwidth is increased by 28.9% relative to the linear one, the gain of the sense mode decreases by 0.37%, and the gain of the drive mode increases by 0.47%. If the peaks spacing is  Δ=230 Hz, the nonlinear bandwidth is increases by 1.76 times, the gain of the sense mode decreases by 0.76%, and the gain of the drive mode decreases by 0.47%. Compared with the peaks spacing at  Δ=560 Hz, the gain of the sense mode is not only improved when the peaks spacing is  Δ=230 Hz, but also the nonlinear micro-gyroscope maintains a fairly wide bandwidth.

It can be clearly seen from Table 2 that for a linear micro-gyroscope, the smaller the distance between the two sense mode peaks, the higher the gain, but the bandwidth decreases significantly. For a nonlinear micro-gyroscope, when the peaks spacing is narrow (such as  Δ=230 Hz), the bandwidth of the sense mode is greatly improved. By comparing it with the linearity, the gain is basically unchanged. It can deal with the problem that the bandwidth must be greatly sacrificed to improve the gain of the linear micro-gyroscope.

The difference between the resonant frequency of the drive mode $\omega_{x}$ and frequency $\omega_{r}\;$ is Δf  $\left(\Delta f=\left(\omega_{r}-\omega_{x}\right)/2\pi \right)\;$. The above analysis demonstrates that the difference Δf  and the peaks spacing Δ have a great influence on the gain and bandwidth. Although nonlinearity has increased a certain bandwidth, it may not be optimal. Taking two structural parameters  Δ=280 Hz and  Δ=230 Hz as illustration, we will optimize the bandwidth and gain of the nonlinear micro-gyroscope in next subsection.

#### 4.3.2. Optimization of Nonlinear Micro-Gyroscope

The relationship between the bandwidth and gain of the nonlinear micro-gyroscope is difficult to be expressed by function. It is a simple and feasible way to use the orthogonal experiment method [20] to design multiple experiments and use the response surface method [21] to fit the bandwidth and gain functions. Taking Δ and Δf  as design variables, and the gain and bandwidth as optimization objectives, the peaks spacing  Δ=280 Hz is optimized. The ranges of the selected variable are 275 Hz≤ Δ ≤ 285 Hz  and 70 Hz≤ Δ ≤ 120 Hz. The data selected by the orthogonal experiment method are shown in Table 3.

Applying the toolbox of MATLAB, the data is fitted with the quadratic regression equation by “regress” instruction. The fitting function is given by:

(28) $$y=a+bx_{1}+cx_{2}+dx_{1}x_{2}+ex_{1}^{2}+gx_{2}^{2}$$

The equations of the response surface model of the bandwidth and gain are expressed as:

(29) $$F_{VK}=-7271.7+51.0952x_{1}+4.3382x_{2}+0.0329x_{1}x_{2}-0.0967x_{1}^{2}-0.0704x_{2}^{2}$$

(30) $$F_{ZY}=-282.5055+0.8918x_{1}+0.1213x_{2}+0.0013x_{1}x_{2}-0.002x_{1}^{2}-0.0017x_{2}^{2}$$

Determine the optimization coefficient of the objective function to avoid errors caused by the data differences. The multi-objective optimization coefficient is given by:

(31) $$\alpha =\frac{\sum_{k=1}^{18}V_{k}/Z_{k}}{18}$$

where $V_{k}$ and $Z_{k}$ are the bandwidth and gain data of group k experiment in Table 3, respectively, and α=−0.535 is obtained. Since two optimization objective functions are involved, they need to be weighed. According to the importance of each objective function, the evaluation function is constructed by:

(32) $$h(F(x))=\sum_{k=1}^{n}w_{k}f_{x}(x)$$

where $w_{k}$ is the weight function and n is the number of objective functions. They should satisfy $w_{k}\geq 0$, k∈(1,n), and $\sum_{k=1}^{n}w_{k}=1$. Taking the weight value as $w_{1}=w_{2}=0.5$ of the bandwidth and gain, the multi-objective optimization function of the micro-gyroscope will be:

(33) $$\left\{ \begin{array}{l} T_{\min}=\frac{w_{2}F_{ZY}-w_{1}\alpha F_{DK}}{w_{1}+w_{2}}\\ s,t\left\{\begin{array}{l} 275\leq \Delta \leq 285\\ 70\leq \Delta \leq 120 \end{array}\right\} \end{array}\right.$$

Use the “fmincon” function in the MATLAB optimization toolbox to solve Equation (33), where “fmincon” is a function for finding the minimum value of a nonlinear multivariate function with constraints. The optimal solutions are as follows:

(34) Δ=279.9358 Hz, Δf=98.2299 Hz

Round the data, i.e., Δ=280 Hz, Δf=98 Hz.

Similarly, when peaks spacing is Δ=230 Hz, the optimization values of the bandwidth and gain are Δ=231.0703 Hz and Δf=75.5711 Hz, and rounded as Δ=231 Hz and Δf=76 Hz. Then, values of the peaks spacing are Δ=280 Hz, 231 Hz, and the resonant frequency of the drive mode $\omega_{x}=5302\times 2\pi \;\mathrm{rad}/s$,  5324×2π rad/s are selected respectively.

**Figure 7:**
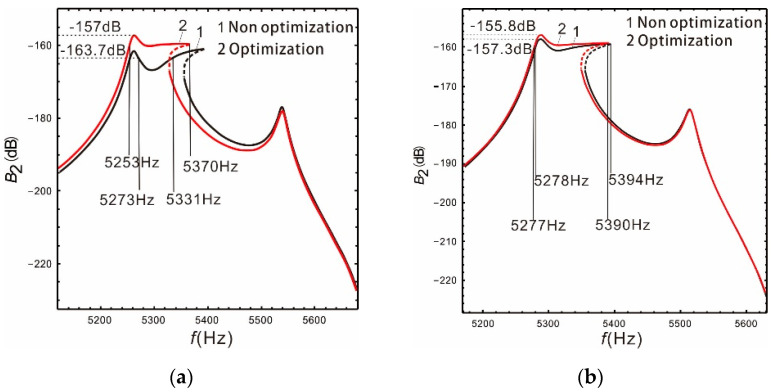
Optimization of bandwidth and gain: (**a**)  Δ=280 Hz; (**b**)  Δ=230 Hz.

As shown in Figure 7a, the optimized bandwidth and gain of the sense mode are 117 Hz and −157 dB, respectively, when the peaks spacing is  Δ=280 Hz. Compared to before optimization, the bandwidth doubled, and the gain increased by 4%. The optimization effect is satisfactory. Figure 7b shows that the optimized bandwidth and gain of the sense mode are 113 Hz and −155.8 dB, respectively. Compared to before optimization, the gain increased by 1.5 dB and the bandwidth decreased by 3 Hz. This is the result of selecting equal bandwidth and gain weights. Obviously, the bandwidth and gain of the nonlinear micro-gyroscope can be further improved by optimizing design. Moreover, the required bandwidth and gain can be obtained by selecting different weight values.

### 4.4. Influence of the Nonlinear Coefficients on the Bandwidth of the Micro-Gyroscope

**Figure 8:**
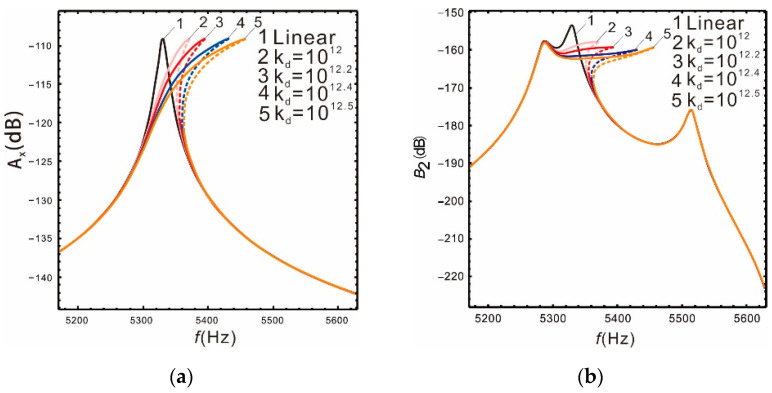
Comparison of the different nonlinear coefficients *k_d_*: (**a**) drive mode; (**b**) sense mode.

Figure 8 is the frequency response of the drive and sense mode under different nonlinear coefficients when the peak spacing is  Δ=230 Hz. As the nonlinear coefficient increases, the bandwidth also increases gradually. See Table 4 for details.

Table 4 shows that when the nonlinear coefficients are $k_{d}=10^{12.5}$, the nonlinear bandwidth of the sense mode is increased by 3.26 times relative to the linear, while the gain is only reduced by 1.08%. In other words, the bandwidth of the micro-gyroscope can be significantly improved by increasing the nonlinear coefficient and the gain is almost constant. However, there are many factors that affect the curve shape of the nonlinear frequency response. For instance, the nonlinear coefficient is too large, the peaks spacing of sense mode is small, or the drive mode resonant frequency is in the middle of the two sense mode frequencies.

**Figure 9:**
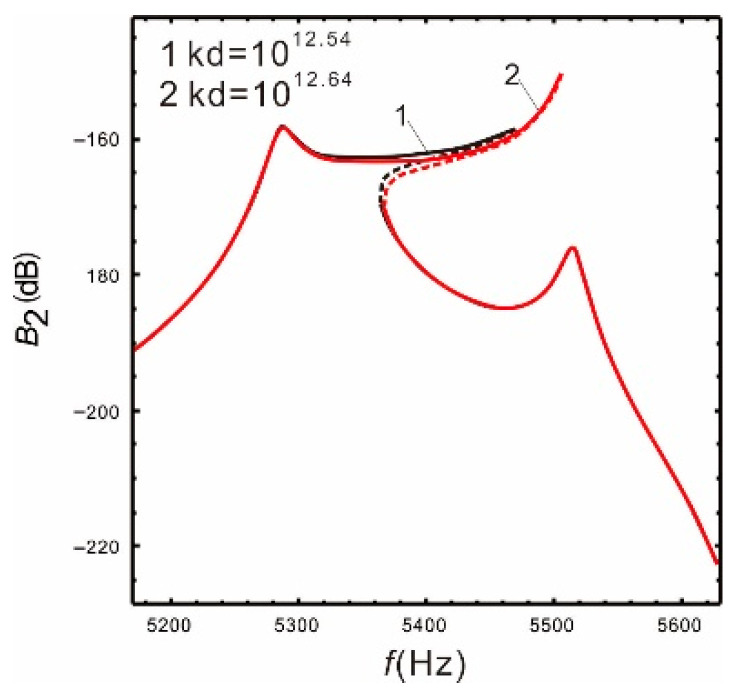
Δ=230 Hz.

**Figure 10:**
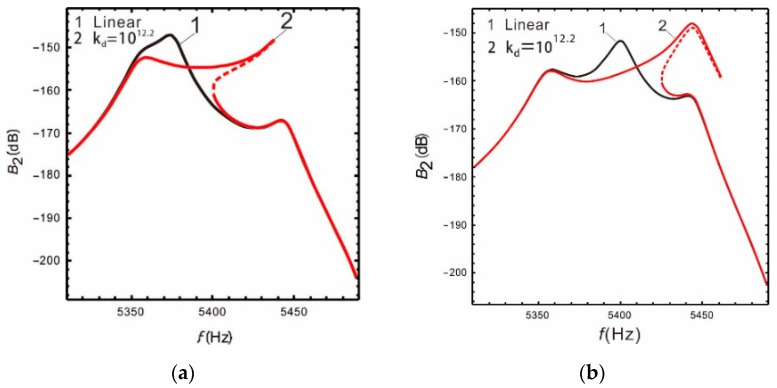
(**a**) $\omega_{x}=5375\times 2\pi \;\mathrm{rad}/s\;,\;\Delta =90\;\mathrm{Hz}$; (**b**) $\omega_{x}=5400\times 2\pi \;\mathrm{rad}/s\;,\;\Delta =90\;\mathrm{Hz}$.

It can be seen from Figure 8 and Figure 9 that when the nonlinear coefficient $k_{d}$ increases to a certain value, the warpage of the response curve suppresses the continuous increase in the bandwidth. Comparing with Figure 10, the warpage of the response curve is also closely related to the peaks spacing. As the peaks spacing decreases, the response curve warps earlier with a smaller coefficient $k_{d}$. Additionally, when the resonant frequency of the drive mode is in the middle of the resonant frequencies of the sense mode, the warpage becomes more obvious. However, the warpage can be avoided by properly selecting the nonlinear coefficients within a given peaks spacing, and the bandwidth of the micro-gyroscope becomes significantly wider at the same time.

### 4.5. Influence of Damping on the Bandwidth of the Micro-Gyroscope

Packaging pressure may change due to different working environments, such as mechanical shock, vibration, and high temperature. The research on the influence of damping on the performance of the micro-gyroscope can provide some references. 

According to the parameter estimation of damping in Section 3.2, the change of package pressure affects the magnitude of damping. In this section, we focus on analyzing the influence of damping on the linear and nonlinear bandwidth of the micro-gyroscope, in which the package pressure increases with the difference of 5 Pa and the nonlinear coefficient $k_{d}=10^{12.2}$ remains the constant in the nonlinear case.

**Figure 11:**
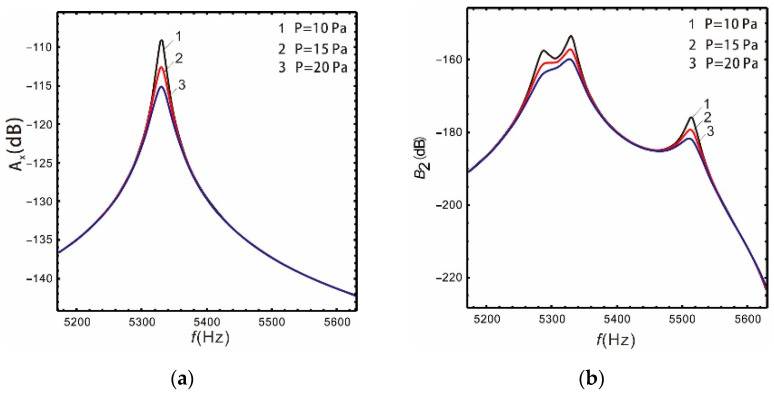
Influence of damping on the linear micro-gyroscope: (**a**) drive mode; (**b**) sense mode.

**Figure 12:**
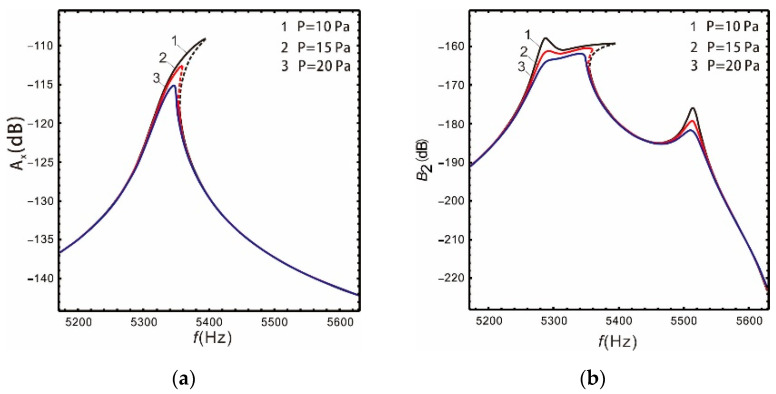
Influence of damping on the nonlinear micro-gyroscope: (**a**) drive mode; (**b**) sense mode.

Figure 11 and Figure 12 show that the gain of the drive and sense mode of both the linear and nonlinear micro-gyroscopes gradually decreases with the increase in damping, and the peak of corresponding resonant frequency decreases sharply. However, the damping has different effects on the bandwidth of the sense mode of the linear and nonlinear micro-gyroscopes.

As shown in Table 5, the increase in damping leads to a reduction in the gain of the sense mode for a linear micro-gyroscope. However, because the peak value is more sensitive to damping, the bandwidth of the linear micro-gyroscope becomes wider with increased damping. When the package pressure *P* equals 20 Pa, it is 19% wider than P=10 Pa. For a nonlinear micro-gyroscope, the increase in damping not only reduces the gain of the sense mode, but also greatly reduces the bandwidth. When the package pressure *P* equals 20 Pa, it is 42.2% lower than P=10 Pa. Through observing the column of sense mode bandwidth in Table 5, with the increase in package pressure, the nonlinear bandwidth increases by 1.76, 0.8, and 0.34 times compared with the linear one, respectively. These results show that the damping has a strong inhibitory effect on nonlinearity. It results in reduced bandwidth and gain, and the frequency response tends to be a linear system.

## 5. Conclusions

This paper investigates a fully decoupled 3-DOF micro-gyroscope based on a complete 2-DOF sense mode system by considering the influence of the Coriolis force. We proposed to improve the performance of micro-gyroscopes by using the hardening nonlinearity of the driving straight beam, and studied the influence of different peaks spacing, damping, and driving nonlinearity on the gain and bandwidth of the sense mode. The main conclusions are summarized as follows:

(1) When the external angular velocity exists, the Coriolis peak in the frequency response of the sense mode produces the same nonlinear hardening characteristics as the drive mode peak. The resonant peaks of the sense mode are not affected by the driving nonlinearity.
(2) The peaks spacing of the complete 2-DOF sense mode system can be adjusted arbitrarily. The smaller the peaks spacing, the higher the gain. When the peaks spacing is narrow, the nonlinearity expands the width of the bandwidth. The generation of nonlinearity slightly reduces the gain compared to linearity, but it can greatly increase the bandwidth.
(3) The bandwidth is very sensitive to the nonlinear coefficient. As the nonlinear coefficient becomes larger, the bandwidth continues to widen. However, the nonlinear coefficient cannot be increased indefinitely, and the value should be selected within a reasonable range.
(4) Large damping can suppress the nonlinearity of the micro-gyroscope. For the linear micro-gyroscopes, increasing damping reduces the gain but the bandwidth increases. For the nonlinear micro-gyroscopes, both the gain and bandwidth are reduced. Therefore, the designed nonlinear micro-gyroscopes should be vacuum packaged as much as possible.

## Figures and Tables

**Table 1:** The parameters of the micro-gyroscope.

Parameters	Values
Thickness of structural layer (*t*)	80 µm
Mass of the drive frame (*m*_b_)	2.85 × 10^−7^ Kg
Mass of the decoupling frame (*m*_p1_)	2.6 × 10^−7^ Kg
Mass of the sense frame (*m*_p2_)	2 × 10^−7^ Kg
Mass of the sense-II (*m*_s_)	1.2 × 10^−7^ Kg
$\mathrm{Number}\;\mathrm{of}\;\mathrm{comb}\;\mathrm{fingers}\;\mathrm{in}\;\mathrm{drive}\;\mathrm{direction}\;(N_{comb}$)	270
$\mathrm{Number}\;\mathrm{of}\;\mathrm{comb}\;\mathrm{fingers}\;\mathrm{in}\;\mathrm{sense}\;\mathrm{direction}\;(N_{cap}$)	500
$\mathrm{Overlapping}\;\mathrm{length}\;\mathrm{of}\;\mathrm{driving}\;\mathrm{comb}\;\mathrm{fingers}\;(l_{comb}$)	40 × 10^−6^ µm
$\mathrm{Overlapping}\;\mathrm{length}\;\mathrm{of}\;\mathrm{sensing}\;\mathrm{comb}\;\mathrm{fingers}\;(l_{cap}$)	16 × 10^−6^ µm
$\mathrm{Gap}\;\mathrm{between}\;\mathrm{the}\;\mathrm{comb}\;\mathrm{fingers}\;\mathrm{in}\;\mathrm{drive}\;\mathrm{direction}\;(y_{comb}$)	10 × 10^−6^ µm
$\mathrm{Gap}\;\mathrm{between}\;\mathrm{the}\;\mathrm{comb}\;\mathrm{fingers}\;\mathrm{in}\;\mathrm{sense}\;\mathrm{direction}\;(y_{cap}$)	4 × 10^−6^ µm
$\mathrm{Mass}\;\mathrm{ratio}\;(\mu^{2}$)	0.26
Proportional parameter (ϵ)	0.97
$\mathrm{Amplitude}\;\mathrm{of}\;\mathrm{the}\;\mathrm{exciting}\;\mathrm{force}\;(F_{0}$)	5.34 × 10^−6^ N
$\mathrm{Damping}\;\mathrm{coefficient}\;\mathrm{in}\;\mathrm{drive}\;\mathrm{direction}\;(c_{b}$)	4.5 × 10^−5^ N·s/m
$\mathrm{Damping}\;\mathrm{coefficient}\;\mathrm{of}\;\mathrm{sense-I}\;(c_{y1}$)	3.4 × 10^−5^ N·s/m
$\mathrm{Damping}\;\mathrm{coefficient}\;\mathrm{of}\;\mathrm{sense-II}\;(c_{y3}$)	1.8 × 10^−5^ N·s/m

**Table 2:** Effect of peaks spacing on gain and bandwidth.

Peaks Pacing (Hz)		Sense Bandwidth (Hz)	Sense Gain (dB)	Drive Gain (dB)
560	Linear	115	−181.6	−137
	Nonlinear	115	−181.6	−137
280	Linear	45	−163.1	−128.2
	Nonlinear	58	−163.7	−128
230	Linear	42	−156.1	−126.6
	Nonlinear	116	−157.3	−127.2

**Table 3:** Data analysis of gain and bandwidth.

Number	X_1_ (Hz)	Δf (Hz)	Bandwidth (Hz)	Gain (dB)	Number	X_1_ (Hz)	Δf (Hz)	Bandwidth (Hz)	Gain (dB)
1	275	70	62	−162.9	10	280	100	113	−157.5
2	275	80	76	−160.7	11	280	110	80	−156.1
3	275	90	111	−158.5	12	280	120	69	−154.8
4	275	100	108	−156.5	13	285	70	60	−164.0
5	275	110	81	−155.5	14	285	80	64	−162.1
6	275	120	63	−154.6	15	285	90	108	−159.8
7	280	70	65	−163.2	16	285	100	115	−157.5
8	280	80	82	−161.3	17	285	110	89	−156.3
9	280	90	109	−159.1	18	285	120	70	−155.2

**Table 4:** Effect of the nonlinear coeffients *k_d_* on gain and bandwidth.

*k_d_*	Sense Bandwidth (Hz)	Sense Gain (dB)	Drive Gain (dB)
Linear	42	−156.1	−126.6
10^12^	96	−157	−127
10^12.2^	116	−157.3	−127.2
10^12.4^	154	−157.5	−127.4
10^12.5^	179	−157.8	−127.5

**Table 5:** Influence of damping on the bandwidth and gain of the drive and sense mode.

Pressure (Pa)		Sense Bandwidth (Hz)	Sense Gain (dB)	Drive Gain (dB)
10	Linear	42	−156.1	−126.6
	Nonlinear	116	−157.3	−127.2
15	Linear	45	−158.8	−126
	Nonlinear	81	−160.6	−124.3
20	Linear	50	−160.2	−124.9
	Nonlinear	67	−162.4	−123.8

## Data Availability

The data presented in this study are available on request from the corresponding author.
